# Host-microbiome interactions and recent progress into understanding the biology of acne vulgaris

**DOI:** 10.1186/s40168-018-0558-5

**Published:** 2018-10-02

**Authors:** Alan M. O’Neill, Richard L. Gallo

**Affiliations:** 1 Department of Dermatology, University of California San Diego, La Jolla, CA 92037 USA; 2 Department of Dermatology, University of California San Diego, 9500 Gillman Dr., #0869, La Jolla, CA 92093 USA

**Keywords:** Microbiome, Acne, *Staphylococcus*, Sebaceous, Inflammation, Commensal, Therapeutics, Skin, Metagenomics, *Cutibacteria*

## Abstract

Acne is one of the most common skin diseases worldwide and results in major health care costs and significant morbidity to severely affected individuals. However, the pathophysiology of this disorder is not well understood. Host-microbiome interactions that affect both innate and adaptive immune homeostasis appear to be a central factor in this disease, with recent observations suggesting that the composition and activities of the microbiota in acne is perturbed. *Staphylococcus epidermidis* and *Cutibacterium acnes* (*C. acnes*; formerly *Propionibacterium acnes*) are two major inhabitants of the skin that are thought to contribute to the disease but are also known to promote health by inhibiting the growth and invasion of pathogens. Because *C. acnes* is ubiquitous in sebaceous-rich skin, it is typically labeled as the etiological agent of acne yet it fails to fulfill all of Koch’s postulates. The outdated model of acne progression proposes that increased sebum production promotes over-proliferation of *C. acnes* in a plugged hair follicle, thereby driving inflammation. In contrast, growing evidence indicates that *C. acnes* is equally abundant in both unaffected and acne-affected follicles. Moreover, recent advances in metagenomic sequencing of the acne microbiome have revealed a diverse population structure distinct from healthy individuals, uncovering new lineage-specific virulence determinants. In this article, we review recent developments in the interactions of skin microbes with host immunity, discussing the contribution of dysbiosis to the immunobiology of acne and newly emerging skin microbiome-based therapeutics to treat acne.

## Background

Acne is one of the most common skin diseases and affects up to 85% of adolescents and young adults worldwide [1]. Severe manifestations of acne are painful and cause disfiguration and scarring, and in some patients, profoundly reduce self-esteem and affects mental health [2, 3]. Acne is considered a disease of the pilosebaceous unit, a complex mini-organ of the body that displays considerable morphological, microbiological, and metabolic diversity depending on the skin site. The sebaceous gland in particular actively responds to fluctuations in hormonal, environmental, and immunological input. Acne development is not only individual-specific but also site-specific, with only some follicles developing inflammation even during severe manifestations of disease. Four main factors are believed to contribute to acne development: increased sebum production, follicular hyperkeratinization, colonization of skin bacteria, and inflammation.

An exciting area of recent advance in the understanding of acne has come from studies focused on the skin “microbiome,” the complex community of bacteria, viruses, and fungal organisms that inhabit all epithelial surfaces and appear to have unique functions on the skin. In sequencing studies of diverse skin sites of healthy adults, the composition of the skin bacterial microbiome was primarily dependent on the particular characteristics and chemical makeup across distinct niches [4–6]. Sebaceous sites were dominated by the lipophilic *Cutibacterium* species (formerly known as *Propionibacterium*), whereas moist areas were abundant in *Staphylococcus* and *Corynebacterium* species. *C. acnes* has been long thought of as a pathogenic factor for acne, yet it is a major skin commensal that prevents colonization and invasion of pathogens, via the hydrolysis of triglycerides in sebum and release of fatty acids that are antimicrobial and contribute to an acidic pH of the skin surface [7].

**Fig. 1:**
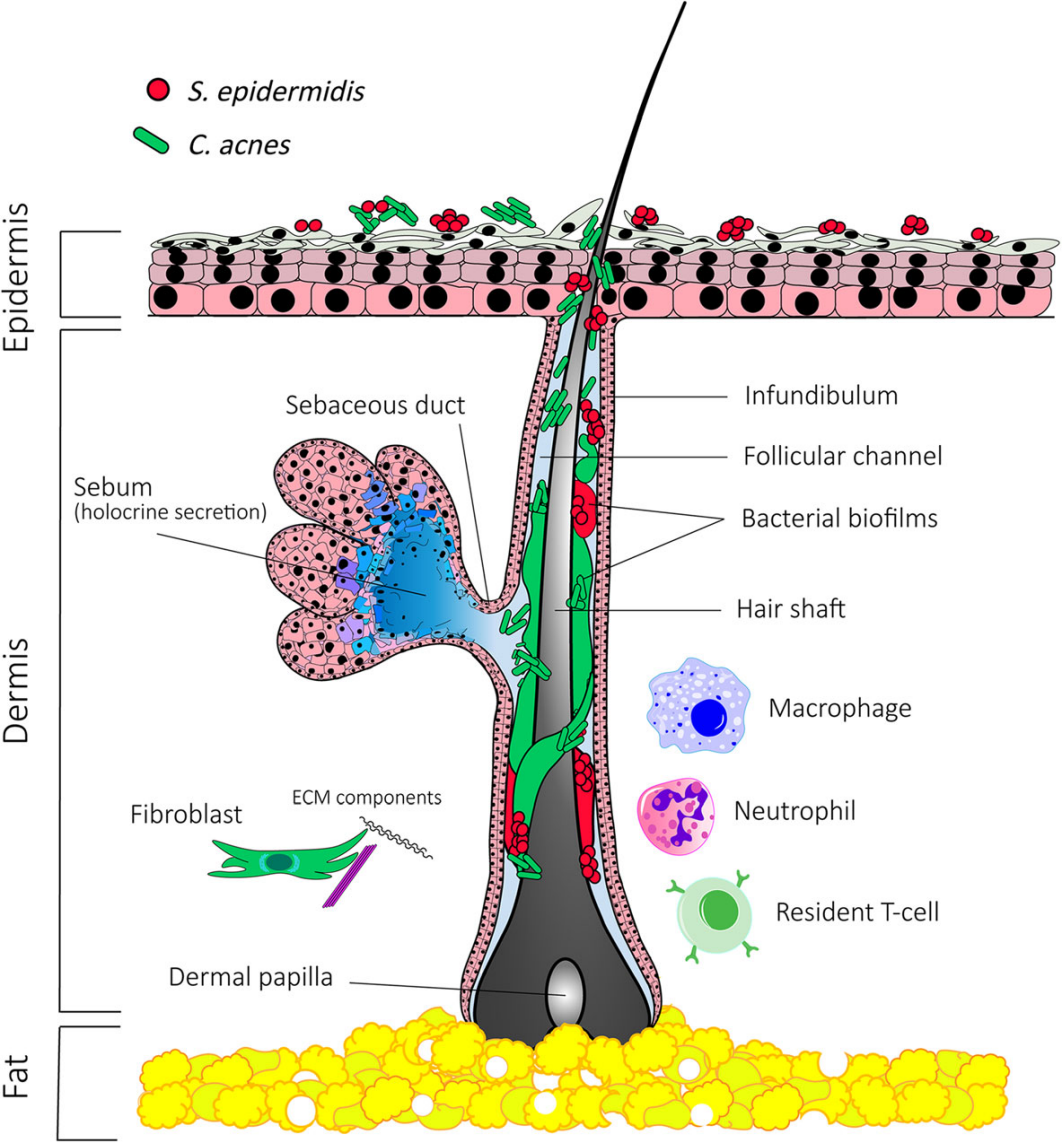
Skin organization and representation of the pilosebaceous unit. Major residents of the pilosebaceous unit, *C. acnes* and *S. epidermidis*, coexist on the skin surface and within the follicle as multiphyletic communities that can interact and coexist

The increasing recognition that commensal and mutualistic microorganisms are necessary for many aspects of normal human physiology has altered the traditional pathogen-dominated view of human-bacterial interactions [8]. The classical assertion that *C. acnes* is the major etiological agent in acne vulgaris is still generally regarded as fact within the medical and lay community. Media advertising of acne-related cosmetic products and prescribing of antibiotics by physicians reinforce the notion of a bacterial origin. To date, some studies and articles still incorrectly attribute acne to “infection” with *C. acnes*, despite its ubiquitous presence on healthy skin [9–11]. Likewise, some graphical illustrations of acne development depict bacteria proliferating in the sebaceous gland [12, 13], yet observations show these structures to be sterile [14]. No convincing evidence exists to suggest bacterial overgrowth corresponds to acne development or disease severity [15–20]. *C. acnes* has been shown to coexist on the skin surface and in the pilosebaceous follicle with other *Cutibacterium* spp., including *Cutibacterium granulosum* and *Cutibacterium avidum*, as well as species belonging to *Staphylococcus*, *Pseudomonas, Corynebacterium*, and the commensal fungi *Malassezia* [21]. However, colonization is by no means universal. Several studies have failed to detect viable bacteria in healthy and diseased follicles [16, 17, 22]. Only recently have researchers developed the tools to visualize *C. acnes* colonization in the in vivo setting and observed biofilms attached to the hair shaft and follicular epithelial wall [23], in some cases extending from the stratum corneum to the base of the follicle (Fig. 1). However, this finding is still much in debate and needs to be confirmed by other groups. *C. acnes* biofilms were reported to be more frequent in acne lesions compared to control follicles [24]. These biofilms can exist as polymicrobial structures containing distinct populations of *Staphylococcus*, *Cutibacterium*, and *Malassezia* [25, 26]. The interspecies interactions in polymicrobial communities may dictate biofilm phenotype, such as enhanced antibiotic resistance and inflammatory capacity. In the pilosebaceous unit, a biofilm matrix can act as a biological glue to physically restrict sebum passage into the infundibulum, leading to comedo formation and/or promote retention and accumulation of corneocytes in the lumen, resulting in a keratinaceous plug and comedone development [27]. The in vitro and in vivo observations of biofilm structures in the follicle need further investigation and by itself support the considerations by many in the field that view acne vulgaris as a chronic disease [28].

In this review, we discuss recent insights into the skin resident microbial communities, including their composition and interactions with the immune system in both health and disease. We also discuss competitive and synergistic interactions between cutaneous microbes and its effect on host immune responses relevant to acne vulgaris. We end by considering important unanswered questions in the field and future research priorities. A greater understanding of host-microbiome interaction in acne is important as interest in targeting the skin microbiome for therapeutic approaches increases.

## The microbiome in acne

The four dominant phyla of bacteria residing on the skin are Actinobacteria, Proteobacteria, Firmicutes, and Bacteroidetes. However, depending on the skin topography and distinct chemical makeup of the site and the individual, the composition and diversity of the resident microbes can differ significantly. In a comprehensive metagenomic analyses of diverse human skin sites, including moist, dry, and sebaceous microenvironments, one study found that individuality and skin topography defined the microbiome composition [6]. Whereas, the strain-level distribution of *C. acnes* was more individual-specific than site-specific, the composition of *S. epidermidis* was less diverse, with a greater dependency on site rather than individual characteristics. In the sebaceous-rich environment, both species are frequently isolated from normal and acne-affected skin [29, 30]. However, given the unique environment and chemistry of the pilosebaceous unit, intrafollicular colonization may not correlate with the surface composition. From studies that have examined the bacterial community structure from pooled samples of extracted follicles, *S. epidermidis* was highly prevalent but much less abundant than *C. acnes* [20, 31, 32]. In contrast, a separate study examined bacterial numbers from several individual follicles and found that *S. epidermidis* counts can be equivalent or higher than *C. acnes* in some follicles [33]. Therefore, while *S. epidermidis* is one of the most dominant species on the skin surface, its contribution to health and follicular disease remains poorly understood.

*Cutibacterium* is consistently one the most abundant genus whose frequency increases as a product of enhanced sebum levels [34, 35]. This *Cutibacterium* enrichment coincides with a decrease in overall microbial diversity and richness. Indeed, *C. acnes* has the metabolic potential to substantially alter its local environment. It contains numerous biosynthetic gene clusters and lipases that together contribute to the production and release of antimicrobial and immunomodulatory molecules [36, 37]. Thus, qualitative and quantitative alterations in sebum during adolescence could have a substantial effect on the microbiome composition via interspecies and host interactions.

Despite the challenge of defining a “healthy” skin microbiome, comparing the microbiomes of diseased and healthy skin could advance our understanding of the mechanisms that can contribute to the pathophysiology of acne vulgaris. Recent studies have associated the presence of certain bacteria with specific disease states. For example, in psoriatic lesions, *Cutibacterium* spp. were underrepresented but *Streptococcus* spp. were significantly more frequent compared to healthy control skin [38]. Likewise, dysbiosis is also a hallmark of atopic dermatitis (AD). *Staphylococcus aureus* (*S. aureus*) colonization on AD skin has been directly correlated to disease severity, but the role of other bacterial members of the skin is unknown [39]. In AD lesional skin, the relative abundance of *Cutibacterium* species is reduced but recovers after successful treatment, suggesting an important commensal role of *Cutibacterium* in skin health.

Much of our current knowledge of *C. acnes* skin colonization is based on the biased application of culture-based methods [40, 41]. Quantitation by colony-forming units (CFU) selects for microorganisms in nutrient-rich artificial growth conditions, unlike the dry and nutrient-poor conditions of the skin, and underestimates the total diversity of the community. In culture-based surveys of the skin, *Staphylococcus* spp. are identified and cultivated more easily than *Cutibacterium* spp.—a slow growing organism that requires hypoxic conditions. Thus, to overcome bias culture practices and capture the true diversity of the bacterial microbiome, researchers have applied advanced sequencing methods to look for variable sequences in otherwise conserved taxonomic markers, like 16S rRNA for ribotyping [20], or the untargeted “shotgun” analyses of the entire collection of genetic elements to obtain the “meta” genome of all resident bacteria [31]. While 16S rRNA sequencing is advantageous to capture genetic diversity in bacterial populations, whole-genome metagenomics provide greater resolution at the strain level by capturing single nucleotide polymorphisms as well as the metabolic profile of microbial communities, which are typically altered in several skin disease states. In a study of the acne microbiome, metagenomic analyses revealed several distinct virulence-associated gene elements that encode for antimicrobial peptides, cytotoxins, and proteases that are enriched in *C. acnes* strains associated with disease [31]. Interestingly, the majority of these genes are localized on several genomic islands as well as a linear plasmid. Nevertheless, it is important to recognize that DNA sequencing approaches have their own set of inherent biases which can be introduced into microbiome datasets at many different stages, from the initial study design to sample collection, storage, and processing as well as the sequencing and complex computational analysis required.

### Sampling methodologies for studying the acne microbiome

The choice of sampling method, anatomical location, and sequencing approach are important factors in any skin microbiome study [42]. The most common methods for sampling the skin microbiome include non-invasive methods such as swab, scrub, and tape stripping that recover microbes residing on the skin surface and within the stratum corneum. These methods have the most relevance for microbiome analysis of superficial skin diseases like psoriasis and atopic dermatitis [38, 39], and when adopted for acne studies, distinct lineages of *C. acnes* were found to be more associated with health or disease [43, 44]. However, acne vulgaris is generally considered a disease of the pilosebaceous unit, and an accurate representation of the acne microbiome may require sampling of the follicular environment. Common “invasive” sampling techniques include pore strips and cyanoacrylate gel biopsy that capture individual hairs or follicular “casts,” respectively. The former approach was utilized by Fitz-Gibbon et al. to characterize the microbiome of acne patients sampled from the follicular contents of the nose [20]. Crucially, to collect enough follicular material for sequencing, the authors pooled all pilosebaceous units within the same skin site, sacrificing sensitivity since uninvolved follicles vastly outnumber acne-affected follicles [45]. Many issues involving bias in sampling methods, anatomical choice, and sequencing were subject of interesting debate following publication of this study [46–48]. In light of such controversies, a recent study compared the skin microbiome of acne patients using three different sampling techniques: swab, pore strips, and gel biopsy combined with multiple sequencing approaches [32]. While greater bacterial diversity was discovered on the surface skin, the overall composition of the surface and follicular environment was comparable for the most abundant species, particularly *C. acnes*, with several strain-types represented in both niches. Overall, the authors concluded that surface or follicular sampling were both suitable approaches for accurate analysis of the skin microbiome in acne research, particularly in the context of *C. acnes* association.

### Different typing methods for *C. acnes*

Early typing methods involving serological agglutination, cell wall sugar analysis, bacteriophage, and fermentation profiling revealed two distinct *C. acnes* phenotypes called type I and II [49–51]. Later, sequence analysis of the housekeeping gene *recA* showed that types I and II represented distinct phylogenetic lineages [52]. An additional phylogenetic group later designated as type III was included after discovery of its distinct long filamentous morphology [53]. Because *C. acnes* has a clonal population structure and a high degree of sequence conservation, multi locus sequence typing (MLST) is required for high-resolution strain typing. MLST is based on sequencing of internal fragments of multiple housekeeping genes, with each different sequence defined as a distinct allele that is then used to generate an allelic profile or sequence type (ST) for every bacterial isolate [54]. Presently, the *C. acnes* type I clade can be subdivided into closely related subtypes: IA1, IA2, IB, and IC, that contain many different clonal complexes (CC) and ST’s. The first MLST scheme developed for *C. acnes*, termed the Aarhus scheme, is based on nine gene loci (MLST_9_) and showed that a single clone ST18 within the phylotype IA1 was globally disseminated and associated with severe acne [44]. Another widely adopted but independent scheme is the MLST_8_ method, an expanded version of the MLST_7_ or Belfast scheme, that could discriminate 285 *C. acnes* isolates into 91 ST’s [43, 55]. This scheme also showed that ST1 (denoted as ST18 by MLST_9_ or ST6 by MLST_7_) of phylotype type IA1 was significantly enriched in acne and that several ST’s from CC72 (type II) and CC77 (type III) were associated with healthy skin. Another group utilized publicly available whole genome sequencing data of all known *C. acnes* strains to devise a single locus sequence typing (SLST) scheme that is advantageous in revealing *C. acnes* ST diversity in mixed microbial communities using pyrosequencing [56]. An alternative single-locus approach was reported by Fitz-Gibbon et al. who used the variable 16S rRNA gene called ribotyping, as well as whole genome sequencing, to demonstrate healthy- and acne-associated ribotypes (RT) in a metagenomic study of acne patients [20]. Evidently, each typing scheme utilizes different loci, and identifying which CC, ST, and/or RT is associated with health or disease can be difficult to determine [57, 58]. Nevertheless, it is now well established that *C. acnes* phylotype IA1 is associated with acne, while types II and III isolates are more commonly associated with healthy skin.

### Metagenomic analysis of acne patients

Fitz-Gibbon et al. reported a similar relative abundance of *C. acnes* between both patient cohorts with the three most abundant ribotypes (RT1, RT2, RT3) evenly distributed among both acne and normal follicles [20]. However, four ribotypes including RT4 and RT5 of the phylotype IA1, (synonymous with ST3 (CC3) and ST4 (CC4) of MLST_8_, respectively) were significantly enriched in 30–40% of patients with acne, but rarely found in individuals with healthy skin. In contrast, RT6 which represents a subpopulation of phylotype II was found to be 99% associated with healthy skin. Interestingly, the non-acne-associated IB, types II and III, are also commonly recovered from deep tissue infections and retrieved medical devices [59]. This could indicate that certain strains can become pathogenic in different environments or simply reflect a likelihood for contamination of these strains given their ubiquitous presence on normal skin [60]. For instance, *C. acnes* type III was not detectable on acne skin but composed approximately 20% of isolates from healthy skin [61]. Therefore, a greater genetic, biochemical, and functional characterization is needed to establish whether type II and III strains can be classified as true commensals and IA1 acne-associated CC’s and ST’s as opportunistic pathogens. If so demonstrated, this would represent a potential new strategy for targeted antimicrobial therapy and a significant advancement for disease diagnostics in distinguishing *C. acnes* contamination or infection in many different medical conditions.

In a recent metagenomic analysis of acne patients, Barnard et al. suggested that their findings of a higher relative abundance of *C. granulosum* in healthy individuals, compared to acne, was evidence for a commensal role [31]. In contrast, early culture-based studies reported that *C. granulosum* is more prevalent in comedones and pustules compared to uninvolved follicles of acne patients [62]. Moreover, *C. granulosum* was reported to demonstrate greater lipase activity compared to *C. acnes* [63]. However, the limited genome data currently available for this understudied bacterium indicates a limited repertoire of virulence-associated genes, with notable absences of Christie Atkins Munch Petersen (CAMP) toxins, sialidases and hyaluronate lyases, thought to contribute to *C. acnes*-host interactions during disease [64]. Thus, further investigations are required to determine the potential health contribution of this minor *Cutibacterium* species.

## Skin microbiome interactions with host immunity

**Fig. 2:**
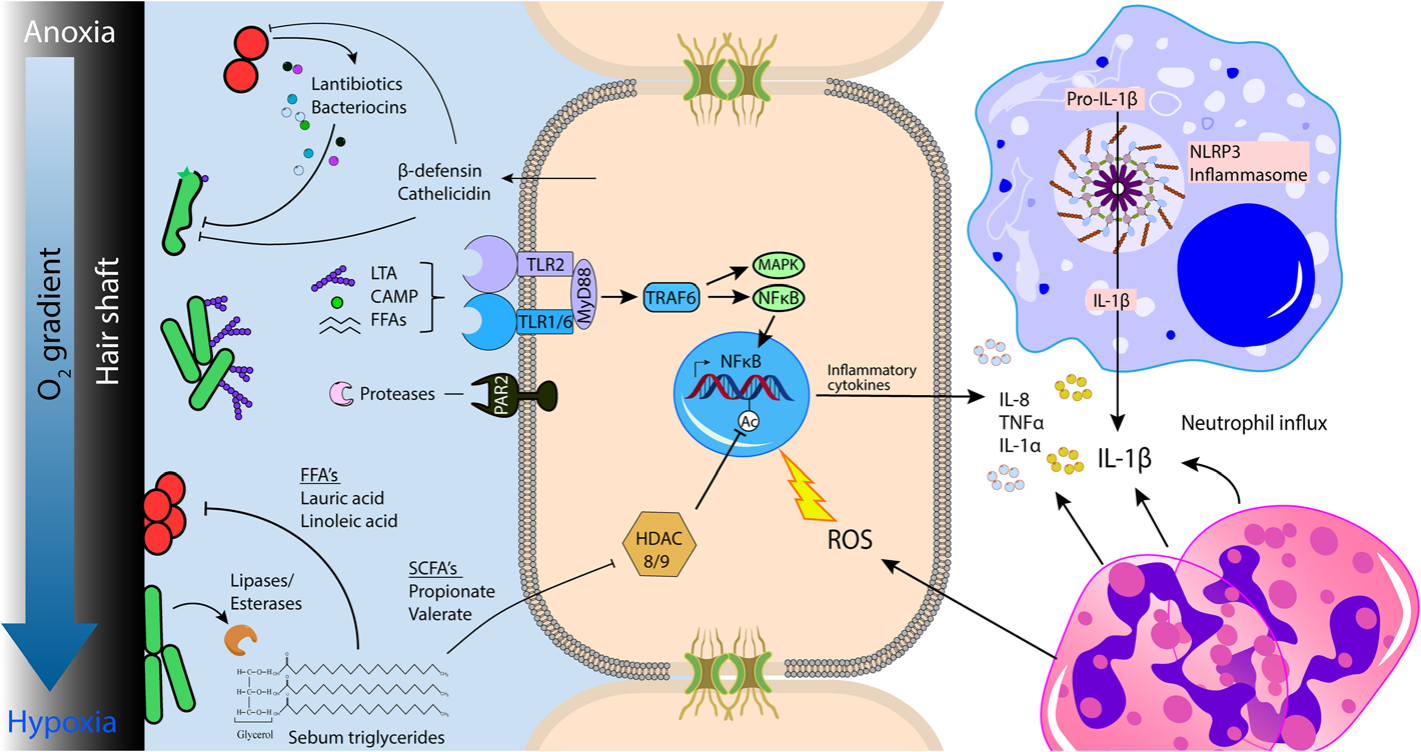
Interspecies interactions and host-bacterial interactions within the follicular microenvironment

Humans have coevolved with their microbiome and developed a wide range of innate immune responses to protect the body against infection, while still maintaining a bacterial presence. In contrast to the gut microbiome that is physically separated from the epithelium by a dense mucus layer in the colon [65], the skin microbiome is in constant and intimate contact with the epithelium, modulating both innate and adaptive immune cell functions [66]. For this reason, it is important that the immune response is primed to recognize and tailored to respond to an appropriate threat, as any immune reaction towards commensals could lead to chronic disease. Keratinocytes are the main cell type of the epidermis and directly participate in both innate and adaptive arms of immunity; as a source of antimicrobial peptides and cytokines that trigger inflammation when the epithelium is exposed to danger- or pathogen-associated molecular patterns (D/PAMP), including Toll-like receptor 2 and 6 (TLR2/6) ligands present on or secreted by many resident bacteria, such as *C. acnes* (Fig. 2) [67]. Such inflammation-causing PAMPs include major components of the coats of gram-positive bacteria such as peptidoglycan (PGN) and lipoteichoic acid (LTA). TLR2 bacterial ligands present on *C. acnes* have been proposed to promote inflammation in acne, stimulating interleukin-1alpha (IL-1α), and granulocyte macrophage-colony stimulating factor (GM-CSF) release [68]. Treatment of keratinocytes with LTA and PGN can trigger NFκB activation via TLR2 activation and release of the neutrophil chemoattractant cytokines TNFα and IL-8 [69]. Early acne lesions and inflammatory papules show significant infiltration of macrophages and neutrophils, which contribute to inflammation and potentially, in sporadic cases, rupture of the follicular wall via the secretion of hydrolytic lysosomal enzymes [70–72]. In one study, it is suggested that the non-acne-associated type II *C. acnes* can induce higher levels of IL-8 in keratinocytes than type IA [73]. In contrast, types IA and IB were found to induce greater levels of the human antimicrobial peptide β-defensin 2 (hBD2) from cultured sebocytes, than a type II isolate [74]. A long-standing question in the microbiome field is why do cells switch from a state of immunological tolerance to a chronic inflammatory state in the absence of an infection? In the case of acne development, a dynamic shift in the microenvironment of the follicle can trigger a different transcriptional response of the microbiota. For example, culturing *C. acnes* in a lipid-rich, hypoxic environment similar to that of an occluded hair follicle, promotes anaerobic fermentation and production of short-chain fatty acids (SCFA) that activates an epigenetic mechanism to enhance the TLR2-mediated production of IL-6, IL-8, and TNFα in human keratinocytes [75].

Colonization by *C. acnes* causes activation of TLR2 in monocytes, resulting in the production of proinflammatory cytokines IL-12 and IL-8. Biopsies of acne lesions also show abundant TLR2 expression on the surface of macrophages surrounding pilosebaceous follicles [71]. In addition, gene expression profiles of inflammatory papules from acne biopsies found marked upregulation of pathways involved in inflammation and matrix remodeling, specifically gene encoding for matrix metalloproteinase 1 and 3 (MMP1, MMP3); β-defensins 1, 2, and 4; and neutrophil granzyme B [76]. There is strong evidence that host antimicrobial peptides (AMP) play a role in the pathogenesis of acne. Skin-derived AMPs comprise the family of β-defensins, S100 proteins, RNases, and the cathelicidin LL-37. While some AMPs are constitutively secreted, hBD-2 and -3 and LL-37 are upregulated in acne lesions and induced by culture supernatants of *C. acnes* in vitro [77, 78].

## Genetic elements of the microbes associated with acne

Since the first *C. acnes* isolate was sequenced in 2004 (KPA171202, a type IB strain recovered from skin), a number of putative virulence genes have been identified with designated functions involved in tissue degradation, cell adhesion, inflammation, and polysaccharide biosynthesis for biofilm formation [79]. Several genetic elements specific to each lineage have since been identified, which could explain the functional differences between lineages and association with different disease states [80]. One of the most fascinating genetic differences between *C. acnes* lineages is the presence of clustered regularly interspaced palindromic repeats (CRISPR)/Cas locus in health-associated type II strains [81]. While this system is only partially present in type III and likely non-functional, it is completely absent from type I strains [82]. CRISPR is a bacterial adaptive immune system against viruses, phages, and foreign DNA, and its presence in *C. acnes* could prevent the acquisition of extra genetic elements that promote virulence and acne pathogenesis [20, 83, 84]. A case in point is the discovery of a novel linear plasmid present in acne-associated type I strains that harbors a tight adhesion (tad) locus, common among many pathogens and essential for biofilm formation, colonization, and virulence [20, 85–87]. Similarly, among the acne-associated strains of RT8, representing clade IB, is a unique genomic island (locus 4) which encode a series of enzymes capable of producing a class of biologically active natural compounds called nonribosomal peptides (NRP) [80]. Other bacteria-derived NRP metabolites have reported roles in cell-mediated toxicity and in iron sequestration as well as potential antimicrobial and antifungal activity [88]. Another plausible genetic explanation for the selectivity of type II strains with healthy skin is the discovery of potentially important mutations in several gene-encoding triacylglycerol lipases, required for the breakdown of sebum [80]. Examining the genetic repertoire of *C. acnes* strains recovered from acne patients revealed several large genomic islands that encode genes homologous with the streptolysin S biosynthetic cluster involved in biosynthesis and transport of bacterial toxins as well as other genes with putative roles in cell survival, virulence, and transport [31]. In contrast, genes which were more abundant on healthy skin were related to carbohydrate and lipid metabolism and nutrient biosynthesis but not virulence. Interestingly, many of these genomic islands that encode putative virulence genes were absent in *C. avidum* and *C. granulosum*. For example, gene-encoding CAMP factors *camp1*, *camp2*, and *camp4*, which have reported roles in hemolysis and inflammation, are absent in both species as well as other genes encoding for host-interacting factors such as hyaluronate lyase and two dermatan sulphate adhesins DsA1 and DsA2—found to be abundantly expressed in the follicular environment [64, 89]. Comparative genomic and proteomic analysis, as well as clinical observations of the three major human *Cutibacteria* in acne pathogenesis, seem to suggest a strict capacity of commensalism for *C. avidum* and *C. granulosum* but a flexibility for parasitism for *C. acnes*. Although it remains unlikely that a single genetic element drives pathogenesis of a multifactorial disease like acne, the absence of suitable tools for the genetic manipulation of *C. acnes* mean that the biological significance of some of these putative virulence genes remain unknown. Alternatively, some groups have tried to identify and characterize the intracellular, membrane-bound, and/or secreted proteome of distinct *C. acnes* strains, to determine their potential contribution to acne pathogenesis [90–93].

## Role of *C. acnes* proteome and interaction with follicular environment

*C. acnes* is considered a contributing factor to the inflammation detected in follicles of patients with acne vulgaris. However, the ubiquitous distribution of *C. acnes* throughout the skin contrasts with the isolated occurrence of inflammatory lesions. The presence of *C. acnes* on healthy and acne skin has often frustrated attempts at assigning a pathogenic role in disease. However, Bek-Thomsen et al. exploited this phenomenon by analyzing and comparing the eukaryotic and prokaryotic proteome of extracted follicular casts from healthy and acne skin [89]. Not surprisingly, acne skin was enriched for host proteins associated with inflammation, wound healing, and remodeling. According to gene ontology analysis, the most significant biological process in acne skin was “response to bacterium.” Interestingly, the only bacterial proteins found belonged exclusively to *C. acnes*. Importantly, the authors also detected less bacterial proteins in acne casts but recorded a shift to a more virulent protein profile, with acne casts enriched for dermatan-sulphate adhesions (DsA1 and DsA2), co-hemolytic toxins CAMP factors 1 and 2 as well as several hydrolytic and lipolytic enzymes. In contrast to reports that attribute *C. acnes* proliferation in acne involvement, the results of this study suggest the existence of a metabolically less-active population in acne, perhaps better adapted to colonize and tolerate the harsh inflammatory microenvironment of an acne-affected follicle. Analogous to the approach adopted by Fitz-Gibbon et al., this study also had several drawbacks including the need to combine extracted casts from both affected and unaffected sites for quantitative analyses. More sensitive approaches are needed to accurately discern the acne proteome of individual follicles [89, 94].

## Bacterial competition within the follicular environment

Interactions between members of the microbiota can shape the resident microbial community by acting competitively to outcompete or eliminate other species or cooperatively for mutual benefits (Fig. 2). In acne, the microbial dysbiosis could be attributed to androgen-mediated seborrhea and dysseborhea, that select for distinct strains which are genetically better adapted to exploit such an environmental shift [95]. For instance, type II strains have been shown to have reduced lipase activity compared to type I strains and could be at a growth disadvantage in a multiphyletic community competing for space and resources [80, 96]. *C. acnes* has been demonstrated to produce several bacteriocins and bacteriocin-like molecules which may be responsible for its successful colonization in the follicle and on the skin surface. Interestingly, the acne-associated phylogroup IA2 display direct antimicrobial activity against a range of *S. epidermidis* strains in vitro [36]. This could be due to the presence of a putative thiopeptide antibiotic, unique to type IA2 strains. *C. acnes* has several defense mechanisms to prevent pathogens that seek to establish colonization in the skin. Indirectly, the metabolic activities of *C. acnes* in sebaceous-rich skin can establish an inhospitable niche against many skin pathogens via the generation of antimicrobial free fatty acids (FFA), including lauric and linoleic acid, as well as SCFAs that can suppress the growth of methicillin-resistant *S. aureus* (MRSA) and group A *Streptococcus* [37, 97]. Similarly, some strains of *S. epidermidis* can ferment carbohydrates to produce the SCFA succinic acid that has potent anti-*S. aureus* and anti-*C. acnes* activity [98].

Genome interrogation of skin *S. epidermidis* strains indicate a vast array of elements involved in interspecies competition. *S. epidermidis* can antagonize *S. aureus* colonization and virulence via quorum sensing signaling—releasing an autoinducing peptide that, when recognized by *S. aureus*, can repress the expression of a global virulence regulator called the *agr* system [99]. Although one study showed strain-dependent antimicrobial activity of *S. epidermidis* against *C. acnes*, it is not yet clear whether acne-associated strains could have enhanced resistance to interspecies killing [36]. However, specific staphylococcal species on the skin secrete lantibiotics with potent antimicrobial activity against *S. aureus* [100]. In fact, the application of these commensal strains onto human subjects with AD was shown to reduce colonization by *S. aureus* [101]. In theory, a similar approach could also be utilized as a potential biotherapy for acne, with application of commensal staphylococcal strains demonstrating broad anti-*C. acnes* activity or even selective against those that are acne-associated. In healthy skin, *C. acnes* and *S. epidermidis* exist as stable heterogeneous communities, but in acne-affected skin, the strain-diversity and relative abundance of *S. epidermidis* is increased at the expense of *C. acnes* [29, 31]. Clearly, a greater understanding of *S. epidermidis* role in acne dysbiosis and pathogenesis is needed and could represent an attractive therapeutic for disease.

## Current treatments that target resident skin microbes

Because acne is a multifactorial inflammatory disease, many different treatment options are available that try to target the underlying causes. Some include topical agents like benzoyl peroxide and salicylic/azelaic acids, anti-androgens, systemic antibiotics, and retinoids as well as physical modalities like laser and photodynamic therapy [102]. Knowledge of the many pathogenic factors is continuously evolving, and better knowledge of disease is crucial for effective management. Isotretinoin is a pro-drug for all-trans retinoic acid and for over 35 years has been prescribed as a “last resort” option for the most severe and recalcitrant manifestations of acne [103]. Yet surprisingly, its mode-of-action is still not fully understood. Its suppressive effect on sebaceous gland activity is well known, and recently, it was reported to normalize aberrant TLR-2-mediated innate immune responses towards *C. acnes* [104]. Logically, isotretinoin should have a major impact on the resident microbe population, by eliminating an essential nutrient supply and stabilizing immune hypersensitivity, but this has not been examined in detail. Interestingly, higher rates of colonization with *S. aureus* have been observed in patients receiving systemic isotretinoin, leading to increased incidence of minor skin infections like folliculitis and furunculosis [105]. Moreover, since relapse rates can be high, occurring in roughly 26–48% of cases [106], elucidating the microbiome composition pre- and post-treatment would be informative. Isotretinoin is a useful treatment to combat acne but has significant drawbacks including, an extended regimen, observational and laboratory monitoring, and some common and serious side effects [103].

The other major treatment options that target the underlying microbial etiology are topical and oral antibiotics. Topical antibiotics act both as antibacterial agents suppressing *C. acnes* and as anti-inflammatory agent. Topical clindamycin or erythromycin is also suitable, and as documented in many randomized, clinical trials, these antibiotics are more effective when combined with benzoyl peroxide (BP) or topical retinoids [102]. BP is an antibacterial agent that targets *C. acnes* through the release of free oxygen radicals and is also comedolytic. Oral antibiotics are indicated for moderate-to-severe disease and for patients in which topical combinations have failed or were not tolerated. Oral antibiotics are taken over a lengthy 3–6-month period [107]. However, the choice for systemic antibiotics to suppress *C. acnes* is counterproductive given the rise of antibiotic resistance [108]. Although it is important to focus on antimicrobial resistance in *C. acnes* during treatment, it is also important to consider the impact of extended antibiotic use on the hugely diverse gut microbiome. The dual approach of topical and systemic therapy does not address the issue of enrichment and transfer of resistance genes in the gut. Moreover, increasing evidence suggests that specific members of the microbiota may never recover after long term antibiotic use. Such alterations in the gut microbiome composition increases susceptibility to infections, particularly recurrent bouts of *Clostridium difficile* [109], increased risk of autoimmune and metabolic disorders like Chron’s disease, irritable bowel syndrome (IBD) as well as type I diabetes, and obesity [110].

Other less-popular alternatives include blue light treatment and conventional UV phototherapy [111, 112]. These approaches may benefit patients with moderate-to-severe acne by altering the skin microbiome and reducing *C. acnes* density in lesions. Indeed, UV-R is known to be bactericidal and can break lipopolysaccharides, LTA, and other bacterial metabolites which have immunomodulatory properties [113].

## Microbiome-based therapeutic approaches to acne

Despite advances in understanding the pathophysiology and immunobiology of acne, no novel products have been brought to market in the last 10 years. Most available treatments are repurposed and still have significant drawbacks. Since 90% of new drug candidates fail to win FDA approval, resulting in average drug development costs of up to $2.6 billion, the apparent dearth of innovation in acne drug development is most likely a feature of its complex etiology [114, 115]. Moreover, to gain regulatory approval, most studies are performed on simplified human skin models under sterile conditions, which fail to accurately model the response in vivo. In the biotechnology and medical sectors, it is now appreciated that microbial dysbiosis in the gut and skin are linked to many chronic diseases and as a result, many new approaches and drug candidates are targeted towards restoring a healthy microbial community. Initial trials of fecal microbiome transplants have been demonstrated to be safe and effective for patients with *Clostridium difficile* infections [116, 117]. Our group is actively involved in therapeutic development of specific bacterial strains selected from the skin microbiome to treat patients suffering with AD. This approach has been shown to eliminate *S. aureus* and restore a balanced microbiome [101]. A similar approach is feasible for acne; however, any microbiome-targeted treatment for acne should consider that the reported dysbiosis relates to distinct *C. acnes* strains and not colonization or infection by a bona fide pathogen per se. Also, new approaches to employ biodegradable nanoparticles or silica microcapsules for delivery within the follicular environment are in development [118, 119]. Better delivery systems can be exploited to achieve better clinical results with classic anti-acne compounds like benzyl peroxide or work in tandem with laser treatment to achieve site-specific release of antimicrobial compounds after activation. We discuss several new microbiome-targeted strategies that are in development or clinical trial stages (Table 1).

**Table 1:** Selected pipeline candidates for the next generation of acne therapeutic drugs

Company	Drug	Formulation	Effect	Mechanism	Stage
Melinta Therapeutics	Radezolid	Topical oxazolidnone	Antibacterial	Second generation antibiotic with enhanced ribosomal binding	Phase II
Allergan and Paratek Pharmaceuticals	Seysara®	Oral sarecycline antibiotic	Antibacterial and anti-inflammatory	Narrow spectrum of activity against *C. acnes* and *S. aureus*	Phase III
Novartis	CJM112		Anti-inflammatory	Anti-IL-17	Phase II
Sebacia	Microparticles	Topical suspension of gold microparticles	Sebostatic	Photothermolysis of sebaceous glands	Phase III
KLOX Technologies	Kleresca®	Cream containing chromophores	Killing of *C. acnes*	Excitation of bacterial porphyrins by light absorption	FDA-approved
Botanix	BTX 1503	Transdermal gel containing synthetic cannabidiol	Anti-inflammatory and sebostatic	Activation of vanilloid-4 ion channels to inhibit sebocyte lipogenesis	Phase Ib
Foamix Pharmaceuticals	FMX101	Topical minocycline foam (4%)	Antibacterial, anti-inflammatory, and antioxidant	Blocks inflammasome-pyroptosis and cleaved IL-1β	Phase III
BiophramaX	BPX-01	Solubilized topical minocycline gel	Antibacterial, anti-inflammatory, and antioxidant	Inhibits protein synthesis in bacteria and inhibits iNOS and MMPs in cells	Phase III
Dermira	Olumacostat glasaretil (DRM01)	Topical small molecule prodrug	Sebostatic	Inhibitor of acetyl coenzyme A (CoA) carboxylase involved in FA biosynthesis	Phase III
Allergan	ANT-1207	Topical botulinum toxin type A	Anti-inflammatory	Acetylcholine inhibitors and glutamate-antagonists	Phase II
Promius Pharma LLC	DFD-03	Topical tazarotene lotion	Anti-inflammatory	Retinoid prodrug: suppress keratinocyte hyperproliferation	Phase III
Celtaxsys	Acebilustat (CTX-4430)	Oral capsule	Anti-inflammatory	Leukotriene A4 hydrolase inhibitors	Phase II
AOBiome	B244	Topical spray	Anti-inflammatory and microbiome modulation	Production of nitrite and NO to suppress inflammation	Phase IIb
Novan Thereputics	SB204	Topical gel	Antibacterial and anti-inflammatory	Release of NO that inhibits IL-1β	Phase III
AndroScience	ASC-J9	Topical cream	Androgen modulation	Synthetic androgen receptor degradation enhancer	Phase III
Galderma	Trifarotene (CD-5789)	Topical cream	Anti-inflammatory	Retinoic acid receptor gamma agonists	Phase III
Seoul National University Hospital	Lupeol	Topical lupeol cream	Suppressed lipogenesis, anti-inflammatory	NF-κB and PI3K/Akt Modulator	In clinical trials
Sienna Biopharmaceuticals	SNA-001	Topical silver particle suspension	Photothermolysis of sebaceous gland	Laser-excited particles that convert light energy to heat	Phase IIb

### Nitric oxide

AOBiome Therapeutics has developed a microbiome-targeted topical and intranasal formulation for the treatment of mild-to-moderate acne. The technology is based on the application of a suspension of *Nitrosomonosa eutropha*, an ammonia-oxidizing bacteria (AOB), isolated from organic soil samples. This therapeutic is reported to exploit the bacterium’s nitrogen cycle to convert ammonia and urea, found naturally on human skin, to nitrite and NO, which have anti-inflammatory and antimicrobial activity. Taking a NO-centric approach, Novan Therapeutics has begun phase III clinical trial for treatment of mild-to-severe acne with a topical nitric oxide-releasing drug called SB204. Results of the phase II trial reported a significant clinical improvement with reductions in number of non-inflammatory and inflammatory lesions [120]. NO exhibits broad antimicrobial activity against many cutaneous pathogens and has a demonstrated role in wound repair [121, 122]. In relation to acne pathogenesis, NO has been suggested to have a dual protective role, directly killing *C. acnes* and also suppressing IL-1β, IL-8, and TNFα cytokine release in keratinocytes [119].

### Probiotic and prebiotics

The first clinical trial evaluating the effects of probiotics on acne was conducted by the physician Robert H. Siver in 1961 using *Lactobacillus* strains [123]. Although he recorded clinical improvement in his subjects, the study crucially lacked a placebo control, leaving Dr. Siver to conclude that, “interactions of skin manifestations of acne vulgaris and of metabolic processes of the intestinal tract are suggestive.” In fact, there is now a scientific merit to this suggestion and is known as the gut-brain-skin axis, which posits a mechanism that connects gastrointestinal health by oral probiotics to skin homeostasis [124]. Recent studies have shown that orally consumed pre and probiotics reduce systemic markers of oxidative stress, inflammation, and insulin resistance and also regulate inflammatory cytokine release in the skin, improving skin barrier function and hydration [125–128].

The concept of bacterial antagonism between *C. acnes* and *S. epidermidis* via fermentation could be applied to develop topical probiotics against acne and other skin disorders. Some skin commensals can produce anti-inflammatory and antimicrobial metabolites under lipid-rich, hypoxic conditions that mimic the follicular environment. *S. epidermidis* can ferment glycerol, a natural constituent of triglycerides in sebum, to produce succinic acid which can inhibit the growth of *C. acnes* and suppress *C. acnes*-mediated inflammation in mice [98]. A feasible approach would be to exploit this phenomenon of interspecies competition to develop a live biologic therapeutic cream containing a rationally selected commensal *Staphylococcus* strain with potent anti-*C. acnes* activity to treat acne lesions.

### Phage therapy

Bacteriophages are viruses that can infect and kill bacteria but are probably the least understood component of the human microbiome [129]. The presence of *C. acnes* phages on human skin was first described over 50 years ago [130, 131], but advances in sequencing technology now provide us with unique insight into the role of viral communities in skin health and disease. Metagenomic analysis has revealed that *C. acnes* phages are more prevalent and abundant in healthy individuals compared to acne patients, consistent with other culture-based studies of *C. acnes* phage counts in acne [20]. Interestingly, a higher relative abundance of phage was detected in older individuals, which could explain why acne prevalence declines with increasing age. To better understand bacterial-phage interactions, Liu et al. challenged genetically distinct *C. acnes* strains with 15 different phages and found that strains from clades IB, II, and III were resistant to killing [132]. This suggested that antiviral strategies of some *C. acnes* strains may shape strain populations in healthy or diseased states, with implications for potential personalized phage-based therapy. Despite phage therapy being utilized in Eastern Europe for over a century and reportedly safe and effective, no data is available on phage therapy specifically for acne treatment [11].

### Vaccines

Vaccination may be a feasible strategy to combat different types of infections caused by *C. acnes*. Potential application of a heat-killed *C. acnes* (HK*Ca*)-based vaccine [133] as well as a vaccine targeting the cell wall-anchored sialidase [134] or secreted CAMP factor 2 have been reported [135]. This approach reduced inflammation and disease severity in a mouse ear infection model. Nevertheless, one of the major obstacles for effective vaccine design is the lack of a suitable in vivo acne model. Moreover, in light of recent metagenomic analysis of acne-associated strains, identifying distinct immunogenic proteins produced by these strains would be an attractive approach. For that purpose, several groups have made recent strides in characterizing the proteome of different *C. acnes* strains [90]. Nevertheless, a *C. acnes* vaccine would have other beneficial effects. For instance, a low dose vaccination with HK*Ca* in an AD mouse model was shown to induce regulatory T cells (Treg) and type 1 T helper (Th1) immune responses, which improved clinical symptoms [136]. Likewise, HK*Ca* was cross-protective against *Actinobacillus pleuropneumoniae* in pig and mouse models [137]. A number of attractive vaccine candidates have been reported from in vivo infection studies, including a rabbit model of implant-associated infection with *C. acnes* that identified 24 immunogenic proteins upregulated during infection [138].

## Animal models for acne vulgaris

In the current system, any new anti-acne drug candidate requires several rounds of screening using biochemical assays, cell culture, and animal models to test for efficacy and toxicity before advancement to clinical trials. Roughly 90% of drugs entering clinical trials fail, and this high-failure rate is partly attributed to a lack of models that accurately represent the complexity and organization of human tissue and/or recapitulate human-microbiome interactions [139, 140]. Therefore, predictive and validated animal models are essential to improve the translation of drug findings from the bench to the clinic. Acne is a complex human disease of the pilosebaceous unit that currently lacks a suitable animal model. Numerous models have been tested, including the Mexican hairless dog, the rhino mouse, and the rabbit ear [141]. The rabbit ear assay is the most common model utilized to determine chemical comedogenicity but does not induce inflammation. Similarly, the hairless rhino mouse is limited as a vaccine model since it failed to produce antibodies against thymus-dependent antigens [142]. Most animals do not develop inflammatory acne-like lesions, and neither *C. acnes* nor *S. epidermidis* naturally colonize murine skin, which is histologically and biochemically different than humans and supports different microbial communities, most commonly *Staphyloccous xylosus* and *Staphylococcus saprophyticus* [143, 144]*.* Animal sebum does not contain sufficient amounts of triglycerides and free fatty acids to satisfy the nutritional requirements for *C. acnes* [145], a fact that has restricted the development of anti-acne drugs and vaccines. Instead, most studies assess sustained “acne-like” inflammation characterized by edema and cell infiltration after intradermal injection of viable or HK*Ca* in the ears of mice. To overcome such limitations, a strategy to mimic the microenvironment of an acne lesion in mice was conducted by constructing a perforated tissue chamber scaffold (to permit influx of immune cells), containing human sebocyte cells and *C. acnes*, to investigate bacterial-host interactions in vivo [146]. Recently, it was proposed that HR-1 mice develop acneiform inflammation and small microcomedone-like cysts after *C. acnes* injection [147]. Alternatively, several ex vivo animal skin models are used to assess the cutaneous permeation of drugs, with porcine skin found to be most histologically similar to humans, in terms of thickness, hair follicle density, and lipid composition [148, 149]. Therefore, because acne is a multifactorial complex human disease, of which colonization of *C. acnes* is a contributing factor, no perfect animal model currently exists. Thus, without a greater focus on developing new models for acne, closing the translational gap will remain a difficult endeavor.

## Human skin models for microbiome studies

Ninety percent of drugs that enter phase I clinical trials eventually fail [150]. Traditional in vitro two-dimensional (2D) cell cultures are the current standard for early drug screening but fail to recapitulate the native 3D microenvironment of in vivo tissues [151]. There are several currently available human skin equivalent (SE) models commercially available,from relatively simple reconstructed epidermis of keratinocytes, to more complex full-thickness skin models containing differentiated epidermis on a fibroblast-embedded dermis matrix [152]. The major technical hurdle of 3D SE models for research and clinical applications is how to integrate endothelial cells, immunocytes, adipocytes, and appendages together with a functional vasculature and innervation component. Nevertheless, one group has successfully incorporated Langerhans cells in a full-thickness human SE that can mount an effective innate immune response to a topical allergen [153, 154]. Likewise, another group recently reported the first SE containing a perfused vascularized network lined with endothelial cells [155]. Another emerging field with potential to revolutionize skin research is 3D bioprinting [156, 157]. A major goal for 3D skin bioprinting is the ability to print tissue that incorporates a structured microenvironment for self-organization of niche cells such as a sebaceous gland, hair follicle, or as recently demonstrated by Liu et al.—morphogenesis of epidermal progenitors to a sweat gland [158]. Given the increasing appreciation that the skin surface and associated appendages are home to a significant biomass of colonizing microorganisms, recent attempts have been made to construct 3D skin models with a microbiome that is more representative of the skin ecosystem. The Leeds model, designed at the Skin Research Centre in the United Kingdom is based on a polymerized fibrin dermal equivalent of primary human fibroblasts overlayed with primary human keratinocytes that formed a slightly acidic, fully stratified epidermis [159, 160]. This skin equivalent (SE) was successfully colonized by *C. acnes* and *S. epidermidis* as well as pathogenic *S. aureus* strains for up to 120 h under dry, real life conditions. Strikingly, while *S. epidermidis* colonization had only a minor effect on gene expression, *S. aureus* modulated the expression of several hundred genes, many involved in inflammation and immune defense [161]. This SE has been commercialized as Labskin® and represents a useful model for investigating microbial and host interactions.

## Conclusions

For over 100 years, studies have investigated the role of *C. acnes* in the pathophysiology of acne vulgaris. To date, this relationship is still very much in debate. However, thanks to recent large-scale sequencing surveys of the microbial communities present on human skin, together with recent metagenomic analyses of the microbiome in multiple skin diseases, we now know that acne vulgaris can be characterized by the dominance of distinct strains of *C. acnes* as well as increases in *S. epidermidis* abundance. However, given some of the limitations and biases of the skin sampling methodologies, further research requires more robust methods and approaches. The ability to obtain reliable and quantitative analyses of the microbiome of lesional follicles from acne patients with different disease severity would represent a major breakthrough in the field. Likewise, a longitudinal metagenomic assessment of the phenotypic changes of the microbiome during treatment with antibiotics or isotretinoin would be hugely informative, particularly in patients that are treatment-resistant or relapse after treatment. Ultimately, increased knowledge of the mutualistic and antagonistic interactions between *C. acnes* and *S. epidermidis* is crucial not only to better understand the pathophysiology, but also to discover secondary metabolites that can be exploited as a therapeutic strategy. While oral and topical antibiotic therapy remains the mainstay treatment approach for acne patients, the indiscriminate targeting of the microbiome and eradication of important commensal species remains a significant drawback. Moreover, the lack of molecular tools to genetically manipulate *C. acnes* together with the absence of a representative in vivo model for acne, represent major obstacles for research in this field.

**Fig. 3:**
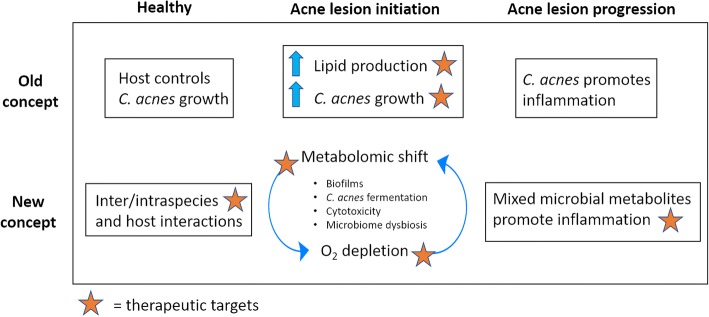
New understandings in the pathophysiology of acne vulgaris

Another exciting area of investigation in the field of acne skin research is the molecular mechanisms that facilitate immune tolerance to colonization by commensal organisms in the skin epithelium and what triggers an inflammatory response in the absence of an infection. We are now beginning to understand how subtle environmental cues can prompt a switch from commensalism to parasitism in *C. acnes* and *S. epidermidis* (Fig. 3). One example is the production of immunomodulatory SCFAs generated during hypoxic conditions. SCFAs are not only antimicrobial in nature, potentially contributing to dysbiosis in the skin, but can also modulate the host epigenetic state that potentiates the epithelium towards an excessive inflammatory response against bacterial TLR ligands that under normal conditions, are well tolerated [75]. Nevertheless, the possibility exists that the microbiome dysbiosis observed in acne disease is predominantly mediated by non-microbial factors such as diet, hormones, or even genetic predisposition. However, the abundance of evidence now showing that distinct *C. acnes* phylotypes and strains induce differential immune responses in multiple cell types, together with new discoveries of novel host-interaction and microbe-interaction genes in *C. acnes* as well as *S. epidermidis*, suggest an important role for these organisms in mediating and promoting acne disease.

## Abbreviations

2D: Two dimensional
AD: Atopic dermatitis
AMP: Antimicrobial peptide
AOB: Ammonia-oxidizing bacteria
BP: Benzyl peroxide
CAMP: Christie Atkins Munch Petersen
CC: Clonal complex
CFU: Colony-forming unit
CRISPR: Clustered regularly interspaced palindromic repeats
Dsa: Dermatan sulphate adhesion
FFA: Free fatty acid
GM-CSF: Granulocyte macrophage colony stimulating factor
hBD: Human beta defensin
HK*Ca*: Heat-killed *C. acnes*
IL: Interleukin
LTA: Lipoteichoic acid
MLST: Multi locus sequence typing
MMP: Matrix metalloproteinas
MRSA: Methicillin-resistant *S. aureus*
NFκB: Nuclear factor kappa B
NRP: Nonribosomal peptides
PAMP: Pathogen-associated molecular pattern
PGN: Peptidoglycan
ROS: Reactive oxygen species
RT: Ribotype
SCFA: Short-chain fatty acid
SE: Skin equivalent
SLST: Single locus sequence typing
Tad: Tight adhesion
TLR: Toll-like receptor
TNF: Tumor necrosis factor
